# A Challenging Case of Coexisting Type A and Type B Lactic Acidosis: A Case Report

**DOI:** 10.7759/cureus.3944

**Published:** 2019-01-23

**Authors:** Monider Singh, Aman N Ajmeri, Mohamed S Suliman, Kamran Zaheer, Amro K Al-Astal

**Affiliations:** 1 Internal Medicine, Joan C Edwards School of Medicine at Marshall University, Huntington, USA; 2 Internal Medicine, Joan C Edwards School of Medicine at Marshall University, Huntington , USA

**Keywords:** case report, lactic acidosis, malignancy, infection, sepsis, type a lactic acidosis, type b lactic acidosis, poorly differentiated metastatic neuroendocrine carcinoma, renal cell carcinoma

## Abstract

The etiology of lactic acidosis can potentially be misleading, especially in a critically ill patient with malignancy. Type B lactic acidosis represents a rare and often lethal complication of malignancy. When differentiating the types of lactic acidosis, Type A is due to marked tissue hypoperfusion and Type B is due to causes in the setting of a normal perfused state.

We report the case of a 56-year-old male with newly diagnosed poorly differentiated neuroendocrine metastatic carcinoma and renal cell carcinoma who presented with a decreased level of consciousness and appetite. The patient was started on a sepsis protocol from an initial intensive care unit (ICU) admission. Broad spectrum antibiotics were initiated, and despite management, his mentation and respiratory status worsened, leading to intubation and mechanical ventilation. The patient continued to have elevated lactic acid and white count levels throughout the hospital course. After extensive workup and an ICU stay of 16 days, a decision was made to pursue comfort care measures and the patient passed away shortly thereafter.

The patient's persistently elevated lactic acidosis may have resulted from the inherent malignancy. The literature mentions glycolysis with enhanced metabolism as a proposed mechanism. One theory states that these changes enable cancer cells to acquire and metabolize nutrients in a way that favors proliferation over efficient adenosine triphosphate (ATP) production, resulting in elevations of lactate production. Patients presenting to the ICU with elevated lactic acid levels need to be thoroughly worked up for all potential causes. In our case, the underlying malignancies likely caused the persistently elevated lactic acidosis, despite subtherapeutic treatment and resuscitative measures.

## Introduction

Lactic acidosis (LA) is a medical emergency; in most clinical settings, its cause is due to marked tissue hypoperfusion (Type A), with sepsis being the most common underlying etiology. Rarely, it can occur in the setting of a normal perfused state referred to as Type B LA [1-3]. LA is an important intermediate in the metabolism of carbohydrates and non-essential amino acids [4]. Lactate is typically the end product of anaerobic glycolysis, but cancer cells can produce a high rate of aerobic glycolysis leading to a substantial amount of lactate [5]. Type B LA is associated with increased glycolysis, increased lactate production by cancer cells, decreased hepatic clearance of lactate, and increased presence of tumor necrosis factor alpha (TNF-𝛼), although the pathogenesis remains unclear [1-2, 6].

In separating the two entities, clinicians need to keep in mind the causes of Type B LA as Type A LA is known to occur in the setting of hypoxia and poor tissue perfusion with the most common causes being cardiogenic shock, cardiopulmonary arrest, severe anemia, and sepsis [3-4, 7]. When considering Type B LA in patients with persistently elevated lactate without evidence of Type A LA features, the differentials expand to underlying liver disease, diabetes mellitus, thiamine deficiency, mitochondrial toxins, seizures, solid and hematological malignancies, or hereditary enzymatic defects [1-3]. This scenario becomes more troublesome when both entities are compounding at the same time and the differentiation becomes blurred due to the present conditions. Herein, we report the case of a 56-year-old male with a newly diagnosed, poorly differentiated neuroendocrine metastatic carcinoma likely from a lower gastrointestinal (GI) source and renal cell carcinoma (RCC) who presented with a worsening level of consciousness and decreased appetite. He subsequently passed away in the hospital with elevated lactic acid and white count levels despite subtherapeutic resuscitation measures.

## Case presentation

This is a case of a 56-year-old Caucasian male who presented to our emergency department at a university hospital with an acutely altered mental status that had declined over 24 hours. He had a decreased appetite the week prior and had been experiencing syncope with falls. Most of the history was, therefore, obtained from the wife. Medical history was significant for poorly differentiated metastatic neuroendocrine carcinoma likely from a lower gastrointestinal (GI) source, renal cell carcinoma (RCC), and gastroesophageal reflux disease (GERD). Pertinent surgical history included recent kidney and liver biopsies consistent with a papillary renal neoplasm, as well as neuroendocrine carcinoma favoring a metastatic process from a lower GI source. Given that these malignancies were recently diagnosed within the past two months, the patient had been on etoposide/carboplatin-based chemotherapy three times a week and had also received filgrastim post-chemotherapy for neutropenia.

On admission, his vitals were stable with a heart rate of 95, SpO_2_ of 95%, respiratory rate of 18, and blood pressure of 113/67 mmHg. An initial arterial blood gas revealed a lactate level of 5.54 mmol/L, bicarbonate of 23.3, and a base excess of 24.4. On physical examination, the patient's mental status was altered and he was markedly confused but alert; however, he was not oriented to time and place. The review of a recent computerized tomography (CT) scan of the abdomen and pelvis showed metastatic liver disease, biopsy-positive for caudal type homeobox Type 2 (CDX 2), synaptophysin, cytokeratin (CK) 20, CK AE1/AE3 (anti-cytokeratin monoclonal antibodies), a right renal mass biopsy positive for CK 7, and racemase, as well as colonic lesions, representing the patient's recently diagnosed malignancies. A non-contrast CT scan of the head was unremarkable on admission. Admission labs revealed a white blood cell (WBC) count of 0.3 k/cmm suggesting neutropenia, a urinalysis positive for trace leukocyte esterase, ionized calcium of 1.67 mg/dL with corrected calcium of 15.6 mg/dL, initial troponin of 0.203, and total absolute neutrophil count of 0.030 k/cmm. Hematology/Oncology Service was consulted, and the patient was subsequently admitted to the intensive care unit (ICU) for further evaluation and treatment.

**Figure 1:**
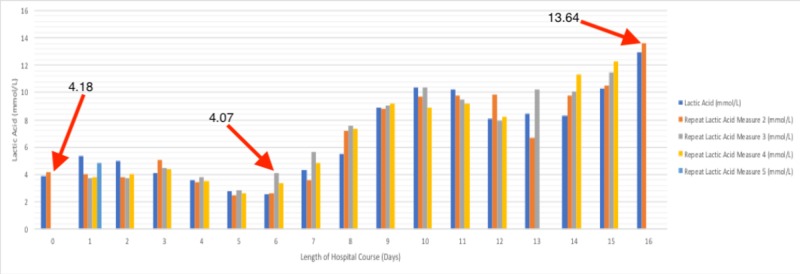
Lactic Acid Trends During Hospital Course There was an initial value of 4.18 mmol/L on Day 0 with fluctuations up to Day 6. At this point, an uptrend started from 4.07 mmol/L and ultimately reached the highest value at 13.64 mmol/L on Day 16.

**Figure 2:**
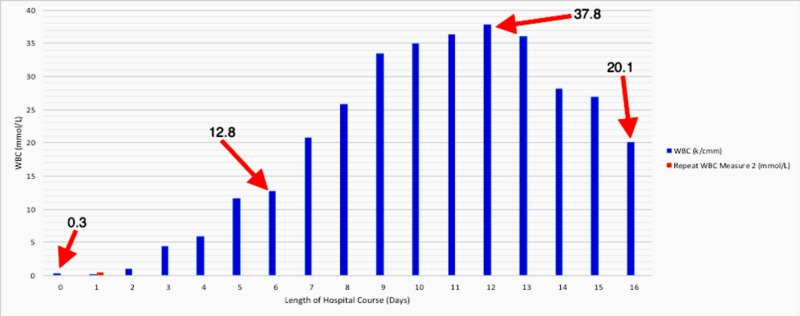
White Blood Cell (WBC) Trends During Hospital Course There was an initial value of 0.3 mmol/L on Day 0 with fluctuations up to Day 6. At this point, an uptrend started from 12.8 mmol/L and ultimately reached the highest value at 37.8 mmol/L on Day 12. From that point, a downward trend started, reaching 20.1 mmol/L on Day 16.

The patient’s assessment included hypercalcemia secondary to malignancy, sepsis - multifactorial, severe neutropenia, and metabolic encephalopathy. Complete workup was done with the initial treatment consisting of pressors (norepinephrine), intravenous fluids (IVF), and zoledronic acid. Blood cultures were positive for gram-negative *Escherichia coli* (E. coli) bacteremia and sputum cultures were positive for *Klebsiella *and *Streptococcus pneumoniae*. The patient was started on vancomycin, as well as meropenem; meanwhile, his condition continued to deteriorate, leading to acute hypoxic respiratory failure requiring mechanical ventilation. Shortly thereafter, his condition started to improve with his lactic acid levels trending down to as low as 2.54 mmol/L and a total absolute neutrophil count trending up to 10.173 k/cmm by ICU Day 5. At the same time, however, his WBC levels were trending up to as high as 11.6 k/cmm. His condition started to hemodynamically decline on ICU Day 6 with the development of fever (100.6°F), elevated total bilirubin (7.5 mg/dL), and increasing lactic acid levels. Infectious Disease Service was brought on board since lactic acid levels were as high as 4.07 mmol/L on ICU Day 6 regardless of appropriate antibiotic coverage (Figure 1). The patient subsequently underwent percutaneous cholecystostomy drainage for possible infectious biliary sludge as well as diagnostic/therapeutic paracentesis removing 3.5 L of fluid, which was ultimately non-pathologic. Despite treating for probable causes of sepsis, by ICU Day 12 the WBC count and lactic acid continued to rise to 37.8 k/cmm (Figure 2) and 9.85 mmol/L, respectively. This prompted the patient’s antibiotic regimen to be changed to linezolid, meropenem, and micafungin.

By ICU Day 13, the patient was hemodynamically stable and afebrile with the lactic acid still elevated at a value around 8.41 mmol/L. In spite of continued elevations in the lactic acid, a spontaneous breathing trial was performed with encouraging weaning numbers. The patient was subsequently extubated and maintained on very low dose norepinephrine, not for hemodynamic stability, but rather to improve end-organ perfusion in hopes that the lactic acid would trend down with optimized antibiotic therapy. The patient's condition was guarded, and the prognosis was poor. On ICU Day 14, the presumed cause of the patient's condition shifted to his underlying malignancies. Despite exhaustive measures, a decision was made after extensive thought and an ICU stay of 16 days to pursue comfort care measures. The patient passed away shortly thereafter with the cause of death presumed to be overwhelming sepsis vs. end-organ failure from underlying malignancies.

## Discussion

Type B LA is infrequently encountered in malignancies and, when present, tends to lead to a poor prognosis as presented in our case [6]. Lactate is commonly used as an indirect marker of illness severity in critical care settings [2]. When a patient is critically ill, the etiology of lactic acidosis can become complicated [6]. Type A LA is associated with oxygen-deficient tissue states, such as sepsis, shock, ischemia, and severe anemia [4]. Type B LA is involved in normal perfused states with the proposed causes being malignancy, thiamine deficiency, alcoholism, liver failure, and seizures [3-4].

To further understand why this type of lactic acidosis is so potentially deadly if not recognized early, one must have knowledge of the pathways involved with lactate. In anaerobic metabolism, lactate is formed via reduction of pyruvate by lactate dehydrogenase (LDH) [2]. This mechanism underlies Type A LA and is present in critically ill patients. Therapeutically, when resuscitative measures and treatments are started, tissue perfusion increases, leading to the lactic acid levels trending down. This may have been present in our patient as the LA trends were decreasing. In contrast, a patient with Type B LA continues to have elevations in lactic acid in an aerobic environment without evidence of prominent causes of Type A LA [2]. One prominent hypothesis is the Warburg effect, noting that tumors and other proliferating or developing cells dramatically increase the rate of glucose uptake, leading to elevations in lactate production from the glycolytic cycle, even in the presence of oxygen and fully functioning mitochondria [1, 8]. The reason for these metabolic changes in a tumor cell is not known, but according to one theory, it is possible that these changes enable cancer cells to acquire and metabolize nutrients in a way that favors proliferation over efficient adenosine triphosphate (ATP) production [3, 9].

Even with the generation of lactic acid, it shouldn’t be enough to cause systemic effects. As reported by Claudino et al., concomitant nutritional deficiencies, decreased clearance due to the liver, and renal impairment may lead to amplification of systemic signs and symptoms [1]. Still, multiple theories have been presented; one postulates the increased presence of TNF-𝛼, which decreases the activity of pyruvate dehydrogenase shunting pyruvate conversion to lactate via LDH [2]. Regardless, the treatment for lactic acidosis is to discern and correct the underlying mechanism producing the lactate, as well as to ensure adequate oxygen delivery in cases of hypoxia. Accordingly, in malignancy-derived lactic acidosis, chemotherapy is the primary treatment modality [6].

With regards to our case, labeling the cause for increasing lactate due to the underlying malignancies could be questioned, along with not initiating chemotherapeutics. Rather than subjecting our patient to low-dose epinephrine, an alternate course of action could have been taken to continue to find a possible infectious source. However, if infectious workup was pursued further without signs and symptoms of infection, it could have possibly led to unnecessary exposure to testing and treatments. At the time, the critical condition of the patient may have dissuaded chemotherapy as the judgment was made in conjunction with the physicians and family involved.

The discussion of a more re-defined treatment approach has been a growing topic. Researchers are targeting the glycolytic pathway even more so now than in the past with the growing number of cases [1]. This would allow for better control of the initial stages of growth and spread by starting at the source pathway rather than trying to mitigate the later stages [1].

With the challenging aspect of having treatable sepsis caused by Type A LA, it can be concluded that our patient had responded subtherapeutically to treatment, but the underlying malignancies were undeniable to not exclude as a cause of the persistent LA. This ultimately led to a turbulent clinical course. Upon review of current literature, there are a relatively growing number of cases with Type B LA and, in particular, a substantial number being caused by solid and hematological malignancies [1-6, 10-11]. This case adds to the growing contingent of literature reporting other malignancies that can cause Type B LA with initial Type A LA leading to a poor prognosis if not treated early in the clinical course [10]. Despite being a well-recognized phenomenon (even though rare), clinical practice should be aimed at the development of rapid diagnosis and improving prognosis [10].

## Conclusions

When a patient presents to the ICU with elevated lactic acid, clinicians tend to conclude it is due to a common set of treatable causes. In our case, the underlying malignancies were causing the persistently elevated lactic acidosis despite extensive treatment for the initial sepsis due to probable Type A LA. Physicians must entertain a diagnosis of Type B LA in any patient with single or multiple malignancies and lactic acid levels unresponsive to long-term resuscitative therapy. Malignancies usually tend to carry a poor prognosis, and with the added component of Type B LA and Type A LA, survival tends to be even poorer. Type B LA is a medical emergency; an aggressive strategy to treat any underlying malignancy, as well as metabolic derangements, once probable causes are treated due to Type A LA may help reduce the mortality and lead to improved prognosis.
